# Rhizobacterial biocontrol of potato late blight and genomic insights into disease suppression and growth promotion

**DOI:** 10.1016/j.isci.2026.117682

**Published:** 2026-09-22

**Authors:** Bekele Gelena Kelbessa, Samrat Ghosh, Farideh Ghadamgahi, Vittoria Catara, Stephen C. Whisson, Rodomiro Ortiz, Ramesh Raju Vetukuri

**Affiliations:** 1 Department of Plant Breeding, Swedish University of Agricultural Sciences, Alnarp, 234 22 Lomma, Sweden; 2 Department of Agriculture, Food and Environment, University of Catania, 95124 Catania, Italy; 3 Cell and Molecular Sciences, James Hutton Institute, Invergowrie, DD2 5DA Dundee, UK

**Keywords:** potato, late blight, *serratia*, biocontrol agent, growth promotion, genome

## Abstract

Late blight caused by *Phytophthora infestans* remains a major challenge to sustainable potato production. In this study, we evaluated five bacterial strains for their ability to reduce late blight and improve potato growth under field, *in vitro*, detached-leaf, and greenhouse conditions. All tested strains reduced disease severity, with *Serratia rubidaea* AV10 showing particularly strong antagonistic activity and consistent suppression of disease. Genome sequencing of the two potato-associated strains, AV10 and *S. rubidaea* EV23, revealed biosynthetic clusters and genes potentially linked to antimicrobial activity, siderophore production, biofilm formation, nutrient acquisition, and plant growth promotion. Bacterial treatment also influenced the expression of potato genes associated with jasmonic acid, ethylene, salicylic acid-related defense, and auxin-linked growth response. Together, these findings identify AV10 and EV23 as promising rhizobacterial candidates for integrated late blight management and provide genomic insight into traits that may contribute to disease suppression and plant growth promotion.

## Highlights

• Bacterial strains isolated from different plants suppress *Phytophthora infestans*
• Potato-associated bacteria harbor putative genes linked to biocontrol and growth promotion
• Strain-specific genomic features may influence the efficacy of late blight suppression
• Bacterial treatments activated defense- and growth-related gene expression in potatoes

## Graphical abstract

**Figure:**
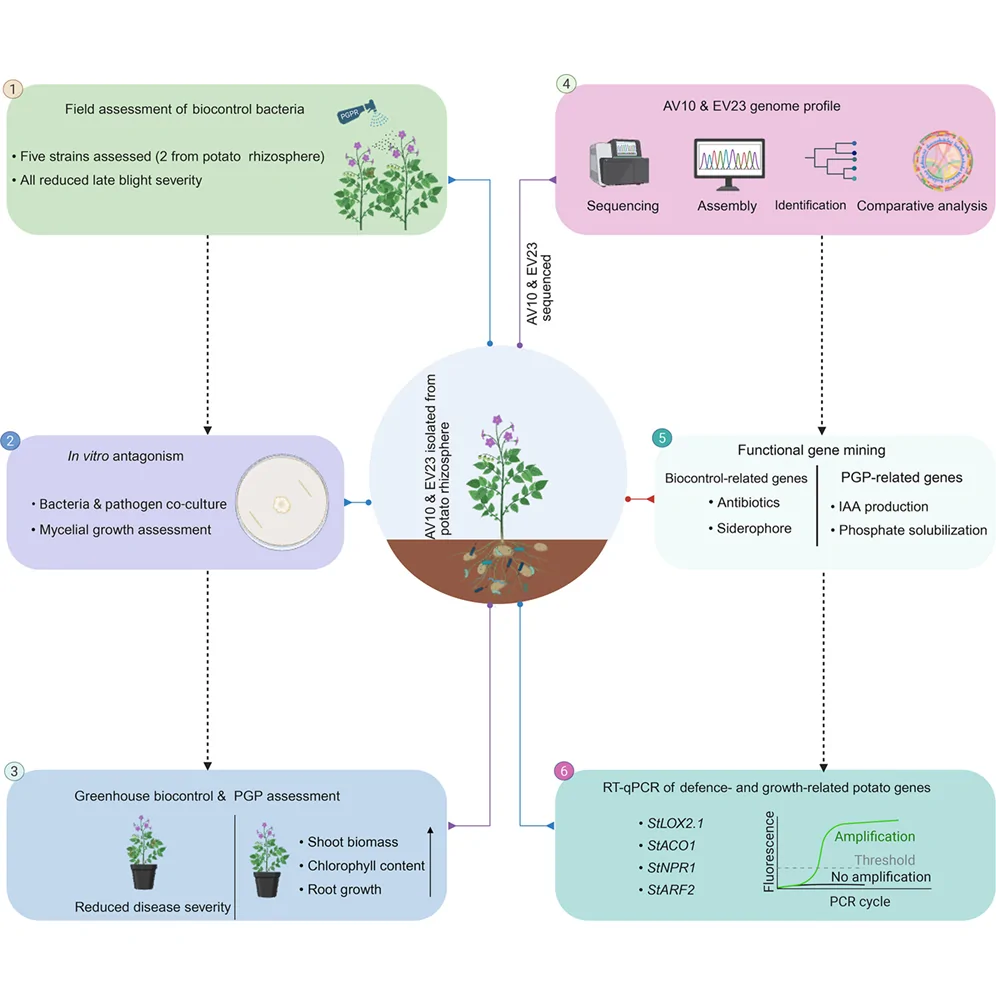

## Teaser

Theoretical nuclear physics; Thermodynamics; Computer simulation; Industrial engineering

## Introduction

Potato (*Solanum tuberosum* L.) is one of the most important non-grain crops for global food security and poverty alleviation owing to its high yields and adaptability to diverse climatic conditions.^1^^,^^2^ However, pests and diseases adversely affect production, causing global yield losses of around 17%.^3^ One particularly impactful disease is late blight, which results from infection of the leaves, stems, and tubers by the oomycete *Phytophthora infestans*.^3^^,^^4^ The costs of managing late blight, in terms of yield losses and increased costs associated with control measures, are estimated at €12 billion per year worldwide.^5^^,^^6^ The costs incurred in any given situation will depend on disease severity, the resistance of the cultivated potato genotype, climatic conditions, and management practices.^7^ *P. infestans* is a hemi-biotrophic oomycete that occurs in all major potato-growing regions and thrives in conditions of low temperatures and high relative humidity (RH).^8^ A potato crop infected with *P. infestans* in an unprotected field can be destroyed within 7–10 days under these conditions.^4^^,^^5^

To mitigate the devastating impact of late blight, various strategies have been implemented, with chemical fungicides remaining the primary measures in potato farming in many countries.^9^^,^^10^ In Europe alone, 39 crop protection products and their co-formulations are registered for the control of *P. infestans* (https://agro.au.dk/forskning/internationale-platforme/euroblight). However, chemical crop protection programs vary considerably across Europe due to differences in climate and disease pressure. For example, cool and humid weather increases disease pressure, leading potato growers in Nordic countries to rely on intensive spraying of preventive crop protectants such as cyazofamid (Ranman) and mandipropamid (Revus).^11^ Although chemical crop protectants are commonly used and effective for controlling late blight, the increasing risks to human health, livestock, and ecosystems associated with their use are raising public concerns.^12^^,^^13^ Such adverse effects are particularly worrisome in situations where potato growers rarely follow recommended fungicide protocols.^14^ Additionally, *P. infestans* exhibits heterothallic reproduction with distinct mating types (A1 and A2). This facilitates sexual reproduction, enabling the emergence of novel genotypes that may be more aggressive and resistant to crop protection chemicals, such as metalaxyl.^15^^,^^16^ Reduced sensitivity to other fungicides may also result from genetic variation among pathogen populations. For example, fluazinam fungicides have been used for many years in Europe to control potato late blight by interfering with energy production in *P. infestans*. However, some clonal lineages in the Netherlands have recently shown reduced sensitivity to this fungicide.^17^ In addition, mutations in the cellulose synthase A3 (CesA3) gene have led to the emergence of *P. infestans* strains resistant to carboxylic acid amide (CAA) fungicides, which target cellulose biosynthesis.^18^ More recently, oxathiapiprolin (OXTP)-resistant *P. infestans* strains have emerged and spread throughout the European population due to mutations in the oxysterol-binding protein (osbp) gene. Reports of combined resistance to several fungicides, including CAA fungicides and OXTP, necessitate updated fungicide-based potato late blight control.^19^

The regulations implemented by the European Union (EU) through Directive 2009/128/EC,^20^ and other increasingly stringent laws on the use of chemical pesticides, also limit the scope for using effective fungicides.^21^

An eco-friendly strategy is thus needed to reduce potato yield losses caused by late blight. Many studies have suggested that treatments using plant-associated microbes could serve as alternatives to traditional pesticides, playing a central role in integrated disease management for sustainable agriculture.^22^^,^^23^ Plant-growth-promoting rhizobacteria (PGPR) are plant-associated microbes that have been studied extensively for their potential to improve the fitness and growth of their host plants directly, indirectly, or through combined mechanisms.^24^^,^^25^^,^^26^ PGPR species can promote plant growth through various direct mechanisms, which may operate simultaneously or sequentially. These direct mechanisms include the solubilization of nutrients (e.g., phosphorus), the facilitation of nutrient uptake by plants, and the production of growth regulators (e.g., auxin).^27^^,^^28^^,^^29^ Indirect mechanisms by which PGPR strains promote plant growth include competing with pathogenic microbes for resources, producing antimicrobial compounds and siderophores, and inducing systemic resistance in plants.^30^^,^^31^^,^^32^

The characterization, development, and use of PGPR strains as biofertilizers and biopesticides for sustainable agriculture have increased in recent decades, aided by advances in genomics and high-throughput sequencing.^33^ These technologies have enabled the identification of genetic traits that promote plant growth and suppress disease in PGPR strains.^34^^,^^35^ The potential of *Bacillus* and *Pseudomonas* strains to promote plant growth and suppress diseases caused by various pathogens across different crops has been studied extensively, and the mechanisms by which they act have been identified.^36^^,^^37^^,^^38^ *Serratia*, a Gram-negative bacterial genus in the Enterobacteriaceae family, has received less attention in this context; only a few studies have explored the potential of *Serratia* strains to suppress disease in potato,^39^^,^^40^ tomato,^41^ and rapeseed.^42^^,^^43^ To further clarify the capabilities of this genus, this study evaluated the biocontrol potential of five bacterial strains: four *Serratia* strains (AS13, S4, EV23, and AV10) and one *Pseudomonas* strain (SLU99) against *P. infestans* under natural conditions to identify strains effective in realistic agricultural settings. Subsequently, *in vitro* and greenhouse experiments were conducted to confirm their disease-suppression activity and evaluate their potential to promote plant growth, both individually and in combination. Some of the selected strains exhibited strong antagonistic effects toward *P. infestans*, demonstrating their potential as biocontrol agents. Thus, the genomes of two newly isolated strains, AV10 and EV23, from the potato rhizosphere were sequenced to identify the genomic capabilities underlying their antagonistic and plant-growth-promoting activities, revealing genetic traits that may be involved in these functions.

## Results

### Bacterial treatments reduce late blight severity under field conditions

Foliar spraying of susceptible potato plants with the selected bacterial strains significantly reduced late blight severity compared with plants treated with the pathogen alone (Figure 1A). Visible infection appeared later in plants treated with these strains, particularly during the active growth stage, with no significant differences among the strains. The strain that resulted in the lowest late blight disease intensity was AV10. However, no strain reduced disease intensity as effectively as fungicide treatment (Revus and Ranman Top) at any time point (Figure 1A).

**Figure 1:**
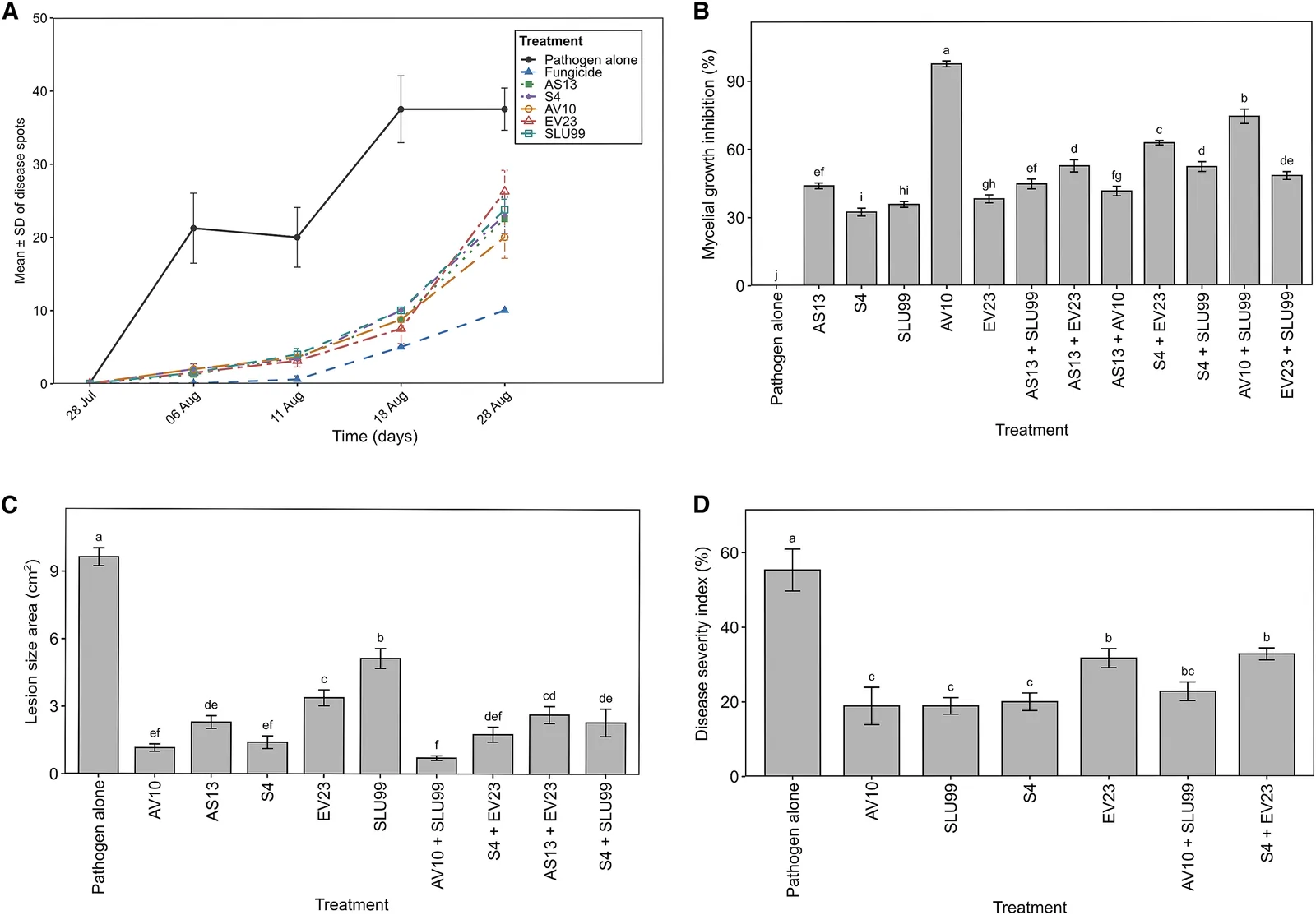
Late blight suppression potential of bacterial strains (A) Mean disease spots of *P. infestans* over five assessment dates (28 July, 6 August, 11 August, 18 August, and 28 August) in the susceptible potato cultivar Desiree after treatment with five bacterial strains, fungicides, and negative controls. Each treatment comprised four replicates (*n* = 4), each with four rows of ten plants. Values represent the mean number of disease spots per block, and error bars indicate standard deviations. (B) *In vitro* inhibition (%) of *P. infestans* mycelial growth by individual bacterial strains and consortia after 14 days of incubation at 20°C, relative to the growth in negative controls without antagonistic bacteria. Strains’ mean inhibitory activities were compared using Duncan’s multiple-range test based on three pooled replicates (*n* = 3), with significance determined at *p ≤ 0.05*. Error bars indicate standard deviation (SD). (C) Late blight lesion area (cm^2^) on detached potato leaves treated with individual or combined bacterial strains, calculated using ImageJ from five pooled replicates (*n* = 5). Data represent the mean lesion area ± standard deviations, with different letters indicate significant difference between treatments according to Duncan's multiple range test at *P ≤ 0.05*. (D) Late blight disease severity (%) on potato leaves treated with single strains or consortia, with four replicates (*n* = 4), six days after spot inoculation with *P. infestans* under greenhouse conditions. Means labeled with different letters differ significantly according to Duncan’s multiple range test at *p ≤ 0.05*.

### All strains inhibited *P. infestans* mycelial growth *in vitro*

*In vitro* evaluation of the chosen bacterial antagonists in dual confrontation assays showed that both individual strains and consortia significantly inhibited mycelial growth of *P. infestans* to varying degrees (Figure 1B and see Figure S1). Strain AV10, which caused the greatest reduction in late blight intensity under field conditions, also exhibited the highest inhibitory effect *in vitro* (97%); the next most effective treatment was AV10 in combination with SLU99 (89.9%).

### Detached leaf assay

The biocontrol effectiveness of the individual strains and consortia chosen for *in vitro* testing was also evaluated by testing their ability to prevent *P. infestans* infection in detached potato leaves. Disease symptoms were assessed by measuring the extent of necrosis caused by *P. infestans*. Treatment with individual strains or consortia significantly reduced the area of necrotic lesions compared with leaves treated with the pathogen alone (Figure 1C and see Figure S2), with the AV10 and SLU99 consortia providing particularly strong protection.

### Biocontrol efficacy of selected strains

The biocontrol performance of selected strains against *P. infestans* was also evaluated under greenhouse conditions. This revealed that the disease severity index (DSI) of potato plants treated with individual bacterial strains or consortia was significantly lower than that of plants treated with the pathogen alone, which had a DSI of 53.34% (Figure 1D). The lowest DSI (15%) was observed with AV10. However, this value did not differ significantly from those for the other tested strains, aside from EV23 and the consortium of EV23 with S4 (Figure 1D).

### General genome characteristics of strains AV10 and EV23

Since *S. rubidaea* AV10 and EV23 both conferred disease suppression and plant growth promotion, but to differing levels, the genomes of these strains were sequenced to enhance understanding of the genomic traits that may contribute to these effects. As shown in Table 1, strain AV10 has a 5 Mb genome with 59% guanine-cytosine (GC) content and 4,761 predicted genes, including 4,608 protein-coding genes, 18 ncRNA genes, 81 tRNA genes, and 11 rRNA genes. Similarly, strain EV23 had a 4.8 Mb genome with 59.31% GC content and 4,558 predicted genes, including 4,432 protein-coding genes. No plasmid was detected in either strain. The genome size of strain AV10 was comparable to that of the reference strain (FDAARGOS926), while EV23 has a slightly smaller genome. Genome maps and summary statistics for both strains are shown in Figures 2A and 2B; Table 1.

**Table 1:** Summary of genome statistics for strains AV10, EV23, and FDAARGOS926

Features	AV10	EV23	FDAARGOS926
Source	potato rhizosphere	potato rhizosphere	clinical
Accession number	JAQOZY010000000	JAQOZX010000000	CP065640
Genome status	draft	draft	complete
Number of scaffolds	5	4	1
Number of chromosomes	NA	NA	1
Completeness (%)	99.88	99.88	99.88
Contamination (%)	0.00	0.45	0.89
N50 [scaffolds] (Mb)	5.0	4.8	5.0
L50 (scaffolds)	1	1	1
Number of contigs	17	22	1
Genome size (Mb)	5.0	4.8	5.0
GC%	59.00	59.31	59.25
Gene count	4,761	4,558	4,697
Protein count (CDS)	4,608	4,432	4,550
tRNA	81	78	84
rRNA	11	11	22
ncRNA	18	13	14
Other	0	0	0
Pseudogenes	43	24	27

**Figure 2:**
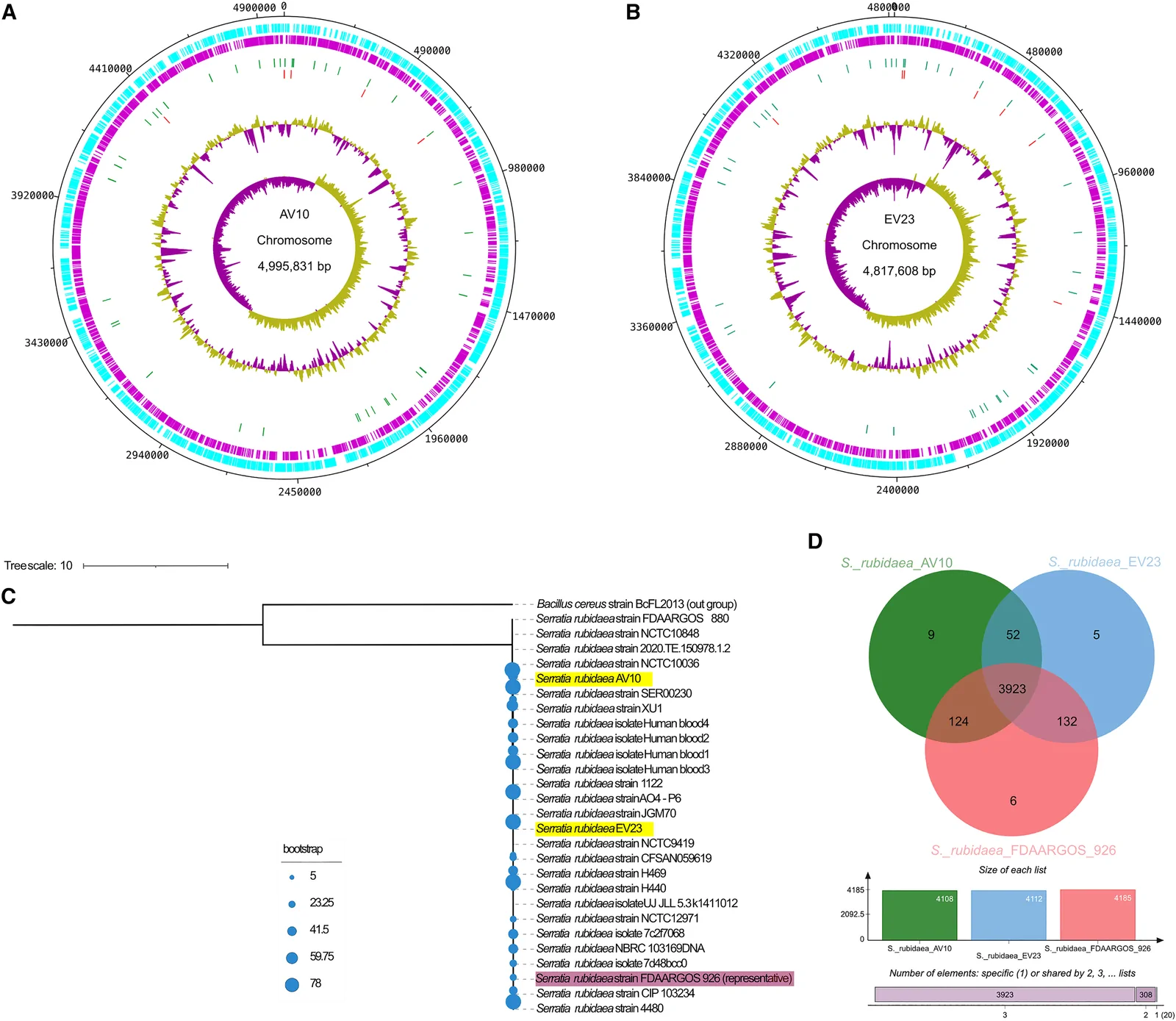
Comparative genomic analysis of *S. rubidaea* AV10 and EV23 (A) Circular maps of the draft genome assembly of the *S. rubidaea* AV10 strain. From the outside to the inner circle, each ring presents distinct information about the genome: coding sequences (CDSs) on the forward (cyan) and reverse (magenta) strands; tRNA genes (green); rRNA genes (red); GC content; and GC skew. (B) Circular maps of the draft genome assembly of the *S. rubidaea* EV23 strain. These maps were generated with DNA Plotter. (C) Phylogenetic tree showing the relationship of *S. rubidaea* AV10 and *S. rubidaea* EV23 (highlighted in yellow) to other *S. rubidaea* strains, and to a non-*Serratia* species (outgroup, highlighted in green), based on 81 bacterial core genes and the encoded gene overlap among AV10, EV23, and the reference strain. (D) Venn diagram showing genes encoded by *S. rubidaea* AV10, *S. rubidaea* EV23, and the reference strain *S. rubidaea* FDAARGOS926. Core genes common to these strains are located at the intersections of the circles.

### Phylogenomic analysis

To determine the taxonomic position of AV10, EV23, and the reference strain, a phylogenetic tree was constructed using 81 bacterial core genes. The pairwise genome comparison indices average nucleotide identity (ANI) and amino acid identity (AAI) were calculated with a cut-off threshold of > 95%, a standard method for delineating prokaryotic species.^44^^,^^45^ The ANI values of the AV10 and EV23 genomes were approximately 98% when compared to the reference strain FDAARGOS926 (see Figure S3; Table S1). The AAI of AV10 was 98.6%, while that of EV23 was 99.3% (see Figure S4; Table S1). These ANI and AAI values (see Table S1) indicate that both strains cluster under *S*. *rubidaea*. Accordingly, a phylogenetic tree based on 81 predefined bacterial core genes (generated with the UBCGv2 pipeline) showed that both strains clustered with other *S. rubidaea* strains (Figure 2C and see Figure S5).

### Comparative genome analysis

Together with other genome characteristics, the number of core genes encoded by strains AV10 and EV23 was compared to that of the reference strain FDAARGOS926. AV10, EV23, and the reference strain share 3,923 protein-coding sequences (CDSs) (Figure 2D and see Table S2). Additionally, 52 genes associated with biological processes are shared between AV10 and EV23 but are absent from the reference strain FDAARGOS926. AV10 contained nine unique genes, of which three were associated with a significantly enriched Gene Ontology (GO) term: ATP binding (GO:0005524). This process is important in the molecular function of energy-dependent processes such as the transport of nutrients and antimicrobial compounds (Table 2). EV23 contained five unique genes associated with two biological processes: glucan catabolism (GO:0009251) and chemotaxis (GO:0006935).

**Table 2:** Summary of significantly enriched GO terms for unique genes in strains AV10 and EV23

Strain	Total genes	GO ID	Name space	GO term name	Count	*p* value
AV10	9	GO:0005524	molecular function	ATP binding	3	0.00461
EV23 5		GO:0009251	biological process	glucan catabolic process	2	0.00018
EV23		GO:0006935	biological process	chemotaxis	3	0.00033
FDAARGOS926	6	NA	NA	NA	NA	NA

The annotated genomes of both strains were subjected to GO and Kyoto Encyclopedia of Genes and Genomes (KEGG) pathway analysis. In strain AV10, Prokaryotic Genome Annotation Pipeline (PGAP) annotated 4,764 GO terms that were grouped into 127 GO-slim categories using CateGOrizer. Further analysis revealed 878 genes associated with molecular functions, 546 with biological processes, and 83 associated with cellular components (see Table S3). In strain EV23, PGAP annotated 4,648 GO terms that were grouped into 127 GO-slim categories using CateGOrizer. This revealed 873 genes associated with molecular functions, 543 with biological processes, and 82 associated with cellular components (see Table S3). Predicted function analysis assigned 3,881 CDS genes in AV10 (84.22% of its total genes) and 3,778 in EV23 (85.24%) to 26 clusters of orthologous groups (COGs) from the COG database, with amino acid transport and metabolism (10%) and carbohydrate transport and metabolism (9%) being the major COG categories (Figure 3A). Transcription and cell wall biogenesis accounted for 9% and 7.4% of the total genes, respectively, in both strains. General functions and predictions accounted for only 4.8%, while unknown functions accounted for 4.3%. Neither strain contained any genes belonging to the COG B group (chromatin structure and dynamics) or the COG Y group (nuclear structure) (Figure 3A).

**Figure 3:**
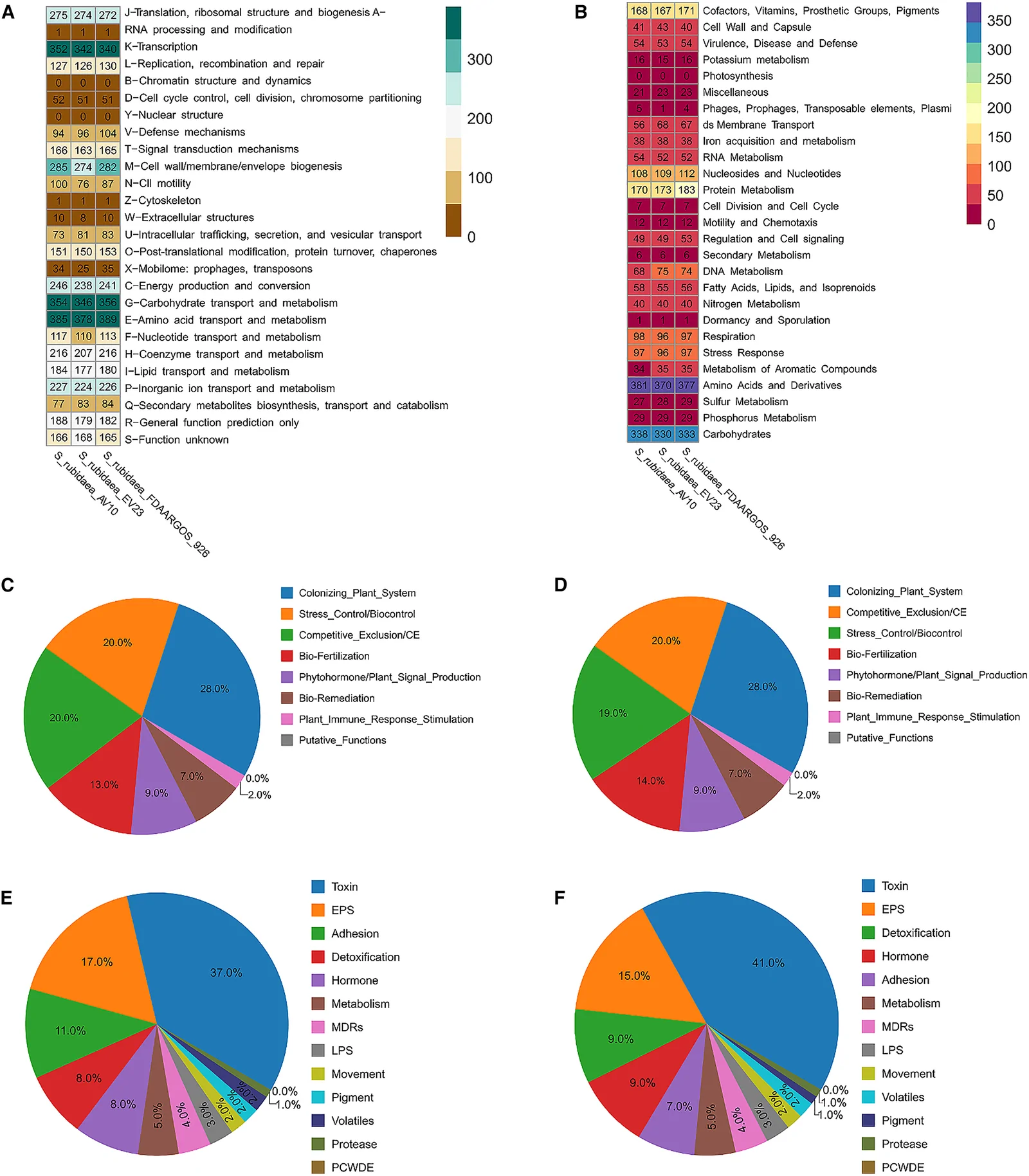
Comparative genomic and functional annotation of *S. rubidaea* AV10 and EV23, including genes associated with disease suppression and plant growth-promotion (A) Distributions of genes annotated with 26 general functional COG classifications. (B) Distributions of subsystem categories showing the main protein-coding genes in the 27 most common subsystem categories. The color scale shows the number of proteins annotated in the COG and subsystem categories. (C) Proportion of coding genes linked to different PGPTs in *S. rubidaea* AV10. (D) Proportion of coding genes linked to different PGPTs in *S. rubidaea* EV23. (E) Proportion of coding genes encoding PIFRs in strain AV10. (F) Proportion of coding genes encoding PIFRs in strain EV23.

Annotation using the KEGG grouped the protein-coding genes into distinct pathway modules, with most (42%) being involved in metabolic pathways. Additionally, 8.5%, 6.7%, and 4.1% of protein-coding genes were involved in carbohydrate, amino acid, and cofactor and vitamin metabolic pathways, respectively (see Table S4). Rapid Annotation using Subsystem Technology (RAST)-based annotation of strains AV10 and EV23 classified their gene functions into 27 different subsystem categories (Figure 3B), mainly involving amino acid and derivative metabolism, followed by carbohydrate metabolism, protein metabolism, and pathways related to cofactors, vitamins, prosthetic groups, and pigments. RAST-based annotation also identified genes involved in disease defense, secondary metabolite production, and stress-related responses that could help plants tolerate abiotic and biotic stresses.

### Prediction of secondary metabolite biosynthesis

Predicted biosynthetic gene clusters (BGCs) in the genomes of strains AV10 and EV23 were detected using antiSMASH. This revealed that the genomes of these strains contained clusters of genes involved in the production of secondary metabolites, including prodigiosin, NRP metallophores, NRPS, NI-siderophores, and aryl polyenes (Table 3).

**Table 3:** Biosynthetic gene clusters for secondary metabolites predicted by antiSMASH in the genomes of AV10, EV23, and the reference strain FDAARGOS926

Strain	Region	Type	From	To	Most similar known cluster (MIBiG)	Similarity (%)
AV10	region 1	prodigiosin	553,696	588,749	prodigiosin	87%
AV10	region 2	NRP-metallophore, NRPS	1,786,219	1,840,761	frederiksenibactin	84%
AV10	region 3	other	2,513,665	2,554,750	pyrrolnitrin	100%
AV10	region 9	NI-siderophore, arylpolyene	4,362,080	4,411,746	APE Ec	73%
EV23	region 2	NRP-metallophore, NRPS	1,711,657	1,765,656	frederiksenibactin	84%
EV23	region 3	other	2,340,303	2,381,388	pyrrolnitrin	100%
EV23	region 10	NI-siderophore, arylpolyene	4,197,469	4,247,141	APE Ec	73%
FDAARGOS926	region 1	NRP-metallophore, NRPS	1	50,854	frederiksenibactin	84%
FDAARGOS926	region 2	prodigiosin	1,232,321	1,267,374	prodigiosin	87%
FDAARGOS926	region 5	arylpolyene,NI-siderophore	2,435,095	2,484,767	APE Ec	100%
FDAARGOS926	region 13	other	4,324,013	4,365,098	pyrrolnitrin	73%

The strains’ genomes also contained genes linked to the production of other secondary metabolites and disease-suppressing proteins, including volatile organic compounds (VOCs) and toxins, hydrolytic enzymes, components of the protein secretion system, and biofilm-forming agents (see Tables S5 and S6).

### Genes associated with plant growth-promoting traits and plant interaction factors

The CDs of AV10 (4,608) and EV23 (4,432) were annotated using PlaBAse to identify genes encoding plant growth-promoting traits (PGPTs) and plant interaction factors (PIFRs) that may contribute directly or indirectly to the strains’ antagonistic or plant growth-promoting activities. Around 28% of the predicted genes (1,290 in AV10 and 1,241 in EV23) are involved in plant system colonization (Figures 3C and 3D; see Table S5). Additionally, 20% of the predicted genes in AV10 (921 genes) were associated with stress tolerance or biocontrol functions, whereas 20% of the predicted genes in EV23 (886 genes) were linked to competitive exclusion (Figures 3C and 3D). The AV10 genome is enriched with a higher proportion of genes associated with stress tolerance and biocontrol activities (20%, 921 genes) than the EV23 genome (19%, 842 genes). Conversely, the EV23 genome harbored a greater proportion of genes related to biofertilization (14%) compared with the AV10 genome (13%) (Figure 3D and see Table S5). Both strains had similar proportions of genes involved in phytohormone biosynthesis, stimulation of plant immune responses, and heavy metal bioremediation. Table 4 lists several plant growth-promoting genes from both strains that are involved in indole-3-acetic acid (IAA) biosynthesis, phosphorus solubilization, and siderophore production.

**Table 4:** *S. rubidaea* AV10 and EV23 genes commonly associated with plant growth promotion traits

*PGPTs*	Genes with potential for PGPTs
IAA production PF02775, PF00205, PF02776, PF00290, PF00291, PF00591, PF02885, PF04715, PF00425, PF00579, and PF01371	*ipdC/ppdC* and trp cluster (*trpA*, *B*, *CF*, *D*, *E*, *S*, and *R*)
Phosphate solubilization PF08042, PF12706, PF03070, PF05402, PF04055, PF13186, PF00675, PF05193, and PF16187	*gcd/gdhAB*, *pqq*, and (*pqqA*, *B/G*, *C*, *D*, *E*, and *F*)
Siderophore production PF12697, PF00975, PF00126, PF03466, PF00501, PF13193, PF00550, PF04055, PF00106, PF13561, PF08659, PF01497, PF03621, PF06276, PF04183, and PF07715	*pchC*, *bauR*, *dhbF*, *hemN/hemZ*, *entA*, *fepB/cbuB*, *mbtH/nocL*, *pmsA*, *rhbC*, and *fepA*

Genes encoding PIFRs were also present in the genomes of both strains. The greatest percentages of predicted PIFR genes were linked to toxin production (37% in AV10 and 41% in EV23), followed by exopolysaccharide substance (EPS) production (17% in AV10 and 15% in EV23) (Figures 3E and 3F and see Table S6). Genes for EPS, detoxification, and adhesion were more prevalent in the AV10 genome. In contrast, genes related to toxin production and hormone signaling were more common in the EV23 genome. Both strains’ genomes contained similar numbers of genes involved in metabolism, multidrug resistance, lipopolysaccharide (LPS) metabolism, motility, volatiles, pigments, and proteases. However, neither genome contained appreciable numbers of genes encoding plant cell wall-degrading enzymes (PCWDEs).

### Bacterial inoculation improves potato growth parameters under greenhouse conditions

The ability of the tested strains to promote potato plant growth under greenhouse conditions was also evaluated. Inoculation of plants with single strains or strain combinations improved plant growth parameters compared with non-inoculated plants (Table 5). The greatest plant height was observed in plants treated with SLU99 (59.86 ± 8.03), followed closely by S4 + EV23 (59.13 ± 4.88); these values were not significantly different from most other treatments except AV10. The highest fresh biomass was recorded in plants inoculated with S4 + EV23 (135.63 ± 8.16), followed by AV10 + SLU99 (129.13 ± 20.79) and SLU99 alone (125.63 ± 10.91). For root weight, the highest value was recorded with AV10 + SLU99 (12.50 ± 5.9), while higher chlorophyll content was observed in plants treated with S4 + EV23 (49.27 ± 4.57). However, for some treatments, growth parameters such as the fresh weight of AV10, EV23, and S4; the fresh root weight of individual strains; and the chlorophyll content of S4 and AV10 + SLU99 did not differ significantly from those of non-inoculated plants.

**Table 5:** Effect of bacterial strains on the growth of potato plants

Strains	Height (cm)	Fresh weight (g)	Dry weight (g)	Fresh root weight (g)	Chlorophyll content (μmol m^-2^)
Control	45.38 ± 4.69^c^	105.88 ± 12.16^c^	7.125 ± 1.46^c^	5.25 ± 1.67^b^	39.83 ± 1.26^c^
S4	55.00 ± 5.13^ab^	122.25 ± 10.94^abc^	10.38 ± 1.85^ab^	10.50 ± 4.04^ab^	43.06 ± 3.58^bc^
EV23	54.19 ± 6.08^ab^	117.13 ± 8.81^bc^	8.50 ± 1.69^ab^	8.00 ± 4.00^ab^	46.48 ± 2.48^ab^
AV10	50.69 ± 2.34^bc^	119.38 ± 11.88^abc^	9.38 ± 1.69^ab^	6.88 ± 3.14^ab^	44.92 ± 3.27^b^
SLU99	59.86 ± 8.03^a^	125.63 ± 10.91^ab^	11.25 ± 1.83^a^	10.38 ± 3.20^ab^	44.96 ± 1.87^b^
S4 + EV23	59.13 ± 4.88^a^	135.63 ± 8.16^a^	10.63 ± 1.06^ab^	11.34 ± 3.74^a^	49.27 ± 4.57^a^
AV10 + SLU99	52.18 ± 4.84^abc^	129.13 ± 20.79^ab^	9.90 ± 1.13^ab^	12.50 ± 5.90^a^	43.65 ± 2.29^bc^

Data are presented as mean ± standard deviation (n = 8), and means were compared using Duncan's multiple range test at *P ≤ 0.05*. Means followed by different letters indicate significant differences among the treatments.

### Bacterial treatment activates defense- and growth-related gene expression in potatoes

To evaluate the role of AV10 and EV23 in regulating plant growth promotion and defense-related genes, the expression of auxin response factor 2 (*StARF2*), *StLOX2.1*, *StACO1*, and *StNPR1* was quantified at 12, 24, 48, and 72 h post-pathogen inoculation. The quantitative reverse-transcription PCR (RT-qPCR) results demonstrated time-dependent and treatment-specific expression patterns. *StARF2* exhibited minimal variation across treatments except for EV23 + pathogen (Figure 4A). A slight induction was observed in the AV10 + pathogen and EV23 + pathogen treatments; however, the EV23 + pathogen treatment showed a transient decrease at an early stage. Expression remained largely stable at 24 h and 48 h, with approximately 2-fold changes observed in pathogen alone (24 h), AV10 (48 h), and EV23 + pathogen (48 h) treatments. At 72 h, *StARF2* expression was increased, particularly in the EV23 + pathogen treatment, followed by the AV10 + pathogen treatment. *StLOX2.1* expression increased at 12 h in AV10 + pathogen and EV23 + pathogen treatments compared with mock and pathogen-only controls (Figure 4B). After a transient decline in expression at 24 h, expression increased again at 48 h in both treatments. At 72 h, *StLOX2.1* expression peaked, particularly in EV23 + pathogen, followed by AV10 + pathogen, indicating extended jasmonic acid (JA) signaling, but the direct causal mechanism remains to be determined.

**Figure 4:**
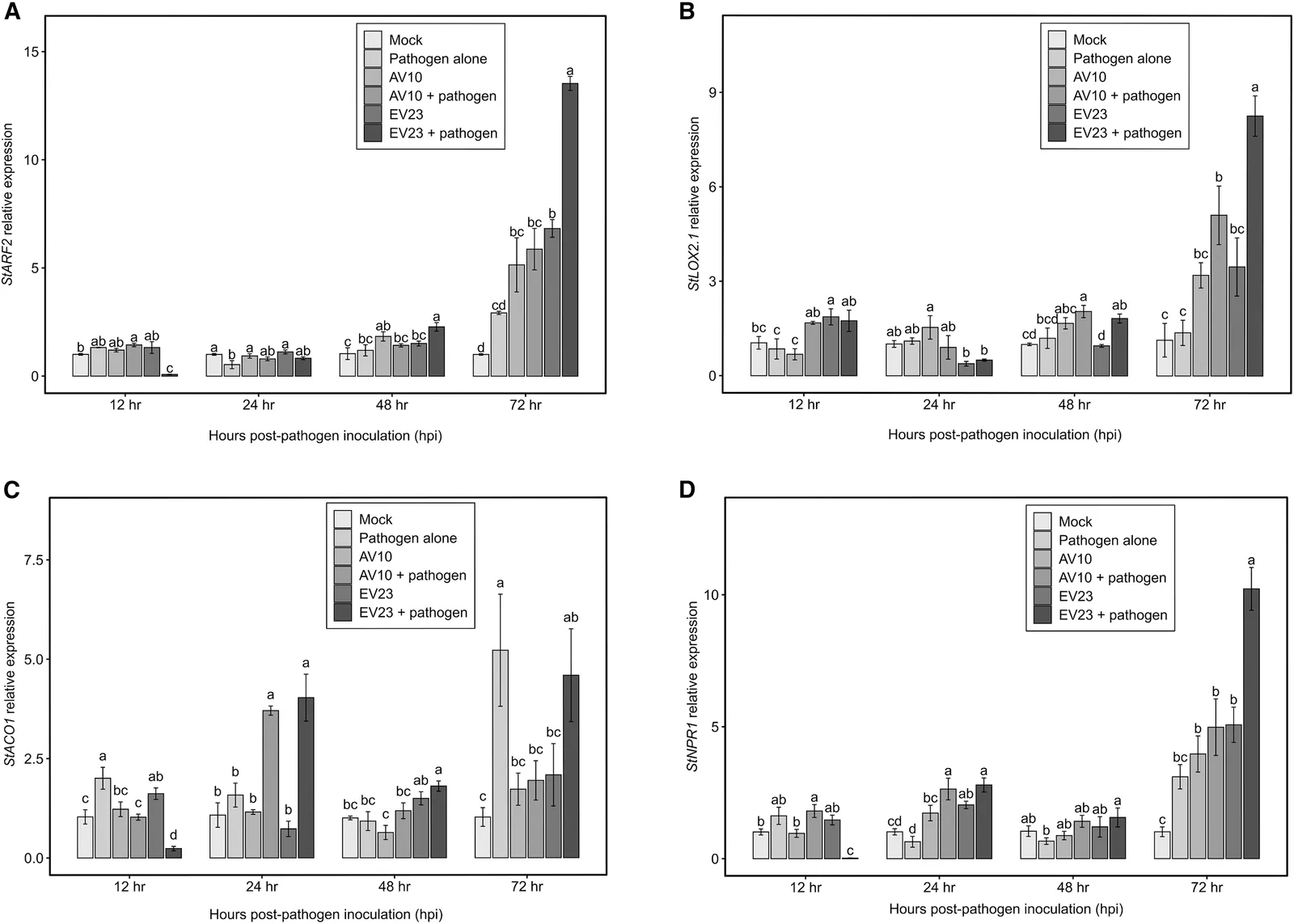
Effect of AV10 and EV23 strains on the expression of auxin and JA/ ethylene (ET) signaling defense response genes in potato plants (RT-qPCR) (A) *StARF2* relative expression. (B) *StLOX2.1* relative expression. (C) *StACO1* relative expression. (D) *StNPR1* relative expression. Data are presented as mean ± SD; treatments with different lowercase letters indicate significant differences between treatments at *p ≤ 0.05* according to Duncan’s multiple range test.

*StACO1* expression was upregulated at 12 h in pathogen- and EV23-treated plants compared with mock, AV10 + pathogen, and EV23 + pathogen treatments (Figure 4C). In contrast, *StACO1* expression was reduced by approximately 4-fold in EV23 + pathogen-treated plants. At 24 h, *StACO1* expression increased in both EV23 + pathogen and AV10 + pathogen treatments, whereas only minor differences (< 2-fold) were observed at 48 h. Expression increased again at 72 h, particularly in pathogen-only and EV23 + pathogen treatments. *StNPR1* expression was reduced at 12 h in EV23 + pathogen-treated plants (Figure 4D), consistent with the expression patterns of *StACO1* and *StARF2*. Expression increased at 24 h in biocontrol agent plus pathogen treatments, which may indicate defense priming, remained relatively stable at 48 h, and reached its highest level at 72 h in EV23 + pathogen-treated plants. The observed expression may require further investigation into potential associations with the salicylic acid (SA)-dependent defense response during pathogen infection.

## Discussion

This study evaluated the biocontrol activity of individual bacterial strains (AS13, AV10, EV23, SLU99, and S4) against *P. infestans* infection under field conditions, as well as their potential to inhibit pathogen growth under controlled conditions both individually and in consortia. Additionally, the genomes of two *S. rubidaea* strains with differing levels of antagonism toward *P. infestans*, AV10 and EV23, were sequenced, and genes associated with various disease-suppressing and plant growth-promoting properties were identified in each. The field trial showed that foliar application of these strains significantly reduced the intensity of *P. infestans* infestation, albeit with lesser efficacy than conventional fungicides. Although plots were separated by 0.5 m and blocks by 1 m, some degree of inoculum movement between neighboring plots cannot be excluded under field conditions and may have reduced treatment differences.

*In vitro*, treatment with the strains individually or in consortia inhibited *P. infestans* mycelial growth and reduced the size of necrotic lesions on detached potato leaves. The AV10 strain showed near-complete inhibition of pathogen mycelial growth under *in vitro* conditions, although its efficacy did not differ significantly from that of most tested strains under greenhouse and field conditions. This inconsistency may result from host-associated and environmental factors that influence its survival, competence in root colonization, and interaction with the plant. It also significantly reduced disease severity under greenhouse conditions, although in this case consortia of multiple strains did not confer greater protection than individual strains. However, both disease control and plant growth were enhanced by combined treatment with strains AV10 and SLU99; the former significantly reduced disease incidence, while the latter promoted plant growth by regulating genes involved in phytohormone biosynthesis.^46^ This aligns with earlier reports showing that treatment with multiple beneficial strains in consortia improves plant resilience under multiple stresses, especially in complex rhizosphere environments, because each strain in the consortium exerts beneficial effects through distinct mechanisms, thereby compensating for the limitations of individual strains and achieving more consistent overall performance.^47^^,^^48^^,^^49^

Treatment of potato plants with AV10 and EV23 activated the expression of JA/ET signaling defense-related genes, which are potentially associated with induced systemic resistance (ISR). Jasmonic acid and ethylene are key plant hormones that regulate the host immune response against interacting pathogens or insect pests, particularly ISR triggered by beneficial microbes.^50^ Increased expression of *StLOX2.1*, a key gene involved in the JA signaling defense response,^51^ following AV10 and EV23 treatments, may contribute to priming a physiological state that helps plants respond rapidly and subsequently suppress pathogen progression. Similarly, increased expression of *StACO1*, which encodes a key enzyme involved in ethylene biosynthesis,^52^ may support the ethylene-signaling defense response in AV10 and EV23-treated plants. Simultaneous increases in *StLOX2.1* and *StACO1* expression in bacteria-treated plants may contribute to transcriptional changes in genes associated with JA/ET signaling pathways. These results suggest that AV10 and EV23 are potentially associated with JA- and ET-related responses during interaction with *P. infestans*. The current study is consistent with many previous studies reporting upregulation of genes involved in JA/ET-signaling defense pathways against pathogens^53^^,^^54^ and nematodes^55^ in plants treated with beneficial microbes. In addition, the expression of *StNPR1* in bacteria-treated plants may indicate the potential role of this gene in defense against late blight. *StNPR1* is a key plant immune regulator that activates the SA-mediated immune response.^56^ The observed increased expression of this gene in bacteria-treated plants suggests activation of the SA-mediated defense pathway. Therefore, the co-expression of JA/ET- and SA-responsive genes may indicate crosstalk between these hormones and network signaling pathways for the defense response.

The suppression of *P. infestans* disease by the tested strains may be related to their putative ability to produce disease-suppressing substances such as hydrolytic enzymes and biofilm-forming agents, and to stimulate host defense genes through ISR. In our previous study, these bacterial strains produced various hydrolytic enzymes (e.g., protease, lipase, and cellulase), formed biofilms, and exhibited PGPTs such as auxin, siderophore, and ammonia.^57^ The AV10 and EV23 genome sequencing in the current study reveals a genetic basis for these phenotypic properties, as the genomes possess genes and pathways associated with disease suppression and plant growth promotion. Similar findings were obtained in a study on the control of *Botrytis cinerea* in strawberries by *S. rubidaea* Mar61-01.^58^ Moreover, *S. rubidaea* S55 was shown to suppress tomato foot and root rot caused by *Fusarium oxysporum* f.sp. *radicis-lycopersici*, both *in vitro* and under greenhouse conditions.^59^

Genome analysis revealed that strains AV10 and EV23 possess multiple genes predicted to be associated with pathogen suppression, including clusters involved in biosynthesis of secondary metabolites such as prodigiosin, NRP metallophores, and NI siderophores. Several reports indicate that *Serratia* strains produce prodigiosin^60^ and other antimicrobial and disease-suppressing secondary metabolites.^61^ They also harbor genes (e.g., *ycgN*, *toxF*, and *yejA*) for the biosynthesis of bactericidal compounds such as carbapenems, toxoflavin, and bacteriocins, as well as genes (e.g., *prnA*, *prnB*, and *prnD*) encoding antifungal pyrrolnitrin enzymes.^62^ These strains also possess genes for the biosynthesis of VOCs that contribute to plant-microbe interactions and antagonism toward pathogens.^63^^,^^64^ Although functional validation has not yet been conducted, the difference observed in disease suppression between AV10 and EV23 under field and *in vitro* conditions may be attributable to their distinct functional gene profiles. Comparative genome analysis showed that AV10 is enriched in ATP-binding genes encoding ATP-binding cassette (ABC) transporter proteins, which facilitate the import of nutrients or the export of antimicrobial compounds. Phenotypically, the AV10 strain also exhibited strong biofilm formation and detectable cellulase activity,^57^ which contribute to surface colonization, persistence, and substrate degradation, respectively. These factors may have contributed to the strong disease suppression observed in strain AV10. In contrast, EV23 possesses genes associated with broad biological processes that may be involved in physiological adaptation rather than in direct suppression of the pathogen. Additionally, the EV23 strain forms weaker biofilms and does not produce cellulase in our previous studies, which may account for its weaker antagonistic effect against *P. infestans*.

The genomes of both strains contain a set of genes (e.g., *trbJ*, *TqsA*, and *IsrG*) associated with quorum-sensing regulation (QSR) and biofilm formation, which may support their colonization of plant roots and provide protection against harmful pathogens.^65^^,^^66^ They also possess genes for flagella biosynthesis (e.g., *flhA*, *flhB*, and *flhE*) as well as genes encoding chemotaxis-related proteins (e.g., *tap*, *tar*, and *tsr*) that may activate signaling pathways that suppress plant pathogens.^67^ A final notable set of genes in these strains encodes one-step (e.g., *hasD*, *comEA*, and *yescC*) and two-step (e.g., *secD*, *secF*, and *secM*) protein secretion systems. These genes may enhance the strains’ competitive and antagonistic activities because Gram-negative bacteria use secretion systems such as T6SS to secrete diverse substances (including effector proteins) into their surroundings, especially during host colonization and pathogenicity.^68^^,^^69^

The genomes of strains AV10 and EV23 lacked a canonical repertoire of PCWDEs with no cellulases, polysaccharide lyases, or carbohydrate esterases identified. However, the AV10 strain encoded AA9-like auxiliary activity proteins and several glycoside hydrolases (GH1, GH31, and GH43). Despite the absence of a canonical cellulolytic gene, these enzymes may facilitate the utilization or modification of plant-derived polysaccharides and may explain previously reported cellulase activity. In contrast, no chitinase activity was observed in plate-assay-based *in vitro* studies in our earlier study, despite the detection of a putative chitinase gene in AV10. Collectively, these findings suggest that genotypic-phenotypic discrepancies may indicate non-canonical enzyme functions or limitations in current functional annotation frameworks. The AV10 and EV23 genomes do, however, contain several genes linked to stress tolerance, including the osmoprotective genes *soxA*, *soxB*, and *soxC* (which promote the accumulation of betaine and glycine) and genes that attenuate salt stress via the accumulation of trehalose and proline, including *treC*, *treR*, and *proA*.^70^ Finally, their genomes contain genes encoding reactive oxygen species (ROS)-scavenging enzymes, antioxidant enzymes, heat shock proteins, heavy-metal resistance, and other universal stress response proteins.

In addition to disease-suppressing traits, both strains possess genes associated with plant growth-promoting properties, including genes for phytohormone production, nutrient acquisition, phosphate solubilization, and siderophore biosynthesis. This is consistent with the observed phenotype, in which the tested strains enhanced potato growth under greenhouse conditions. For example, the StARF2 in plants treated with bacteria may indicate tryptophan (trp)-dependent IAA production. The sequenced genomes of these bacterial strains also contained several genes associated with phytohormones, including *ipdC*, which encodes indole-3-pyruvate decarboxylase, a key enzyme in the trp-dependent biosynthetic pathway, as well as genes associated with cytokinin biosynthesis and transport, such as *miaA*, *miaB*, and *miaE*.^71^^,^^72^

Several studies have demonstrated that certain bacterial strains increase phosphorus bioavailability by solubilizing insoluble forms of phosphorus via gluconic acid production mediated by glucose dehydrogenase.^73^^,^^74^^,^^75^ The genomes of both strains contain the *gcd* gene and the PQQ locus (*pqqA-F*),^76^ along with other phosphate transporter genes (e.g., *pstA*, *pstB*/*phoT*, and *pstC*/*phoW*) and genes involved in regulating homeostasis (e.g., *phoB*, *ppk*, and *phoH*), suggesting that they may promote plant growth by solubilizing phosphate and altering its transport and homeostasis within the host plant.

Siderophores are small iron-binding compounds that help PGPR strains supply Fe to plants and promote their growth,^77^ or inhibit pathogens that cause plant disease.^78^ The genomes of strains AV10 and EV23 contain several genes (e.g., *bauR*, *dhbF*, and *hemN*/*hemZ*) linked with the biosynthesis of siderophores, including pyochelin, acinetobactin, bacillibactin, and mycobactin. EV23 also possesses the *pchC* gene, which is associated with pyochelin production via non-ribosomal peptide synthases.^79^ In accordance with earlier *in vitro* results,^57^ their genomes carry several genes encoding proteins that produce these growth-promoting factors. They also harbor genes associated with atmospheric nitrogen fixation, potassium solubilization, sulfur assimilation, and the metabolism and transport of plant root-derived carbonaceous compounds, including amino acids, carbohydrates, hydrocarbons, nucleotides, nucleosides, organic acids, and other exudates. These findings suggest that the tested strains can suppress microbial pathogens such as *P. infestans* via several different and complementary mechanisms. In conclusion, all tested bacterial strains, individually or as consortia, suppressed potato late blight and improved potato plant growth. The observed disease suppression may be associated with beneficial traits predicted from genomic analyses and previously reported phenotypes, including hydrolytic enzyme production, biofilm formation, stimulation of host defense genes, and the production of putative secondary metabolites, antibiotics, and VOCs. Although the exact mechanisms involved remain to be determined, these and other traits may contribute directly or indirectly to reducing late blight and improving plant growth. Overall, these findings suggest that integrating these bacterial strains with other disease management strategies could effectively reduce late blight damage, thereby reducing reliance on synthetic crop protection chemicals.

### Limitations of the study

Although the tested strains have the potential to suppress *P. infestans* and enhance plant growth, further research is required to assess their effectiveness against the pathogen in multi-location and multi-season trials. Additionally, conducting dose-dependent experiments to evaluate these biocontrol agents will provide insight into their efficacy against late blight and help optimize them. The relative expression of potato genes after treatment with the AV10 and EV23 strains and subsequent pathogen challenge provided insight into transcriptional responses associated with growth promotion and JA/ET signaling defense pathways. Additional studies on hormone quantification, metabolomics, and protein-level interactions should be conducted to strengthen these mechanistic insights. The molecular mechanisms by which AV10 and EV23 directly inhibit *P. infestans* or promote plant growth remain to be elucidated; approaches such as random mutant screening for loss of activity against *P. infestans* or targeted knockout of AV10-specific genes could help reveal them. Further research is also needed on their long-term field efficacy, rhizosphere colonization competence, effects on native microbial communities, and biosafety, as some *Serratia* strains may act as opportunistic pathogens.

## Resource availability

### Lead contact

Requests for further information and resources should be directed to and will be fulfilled by the lead contact, Ramesh Raju Vetukuri (ramesh.vetukuri@slu.se).

### Materials availability

Bacterial strains used in this study are available upon request from lead contact. This study did not generate new, unique plasmids, or reagents.

### Data and code availability

• Assembled genomes and the corresponding raw Illumina reads for *S. rubidaea* EV23 (GeneBank: JAQOZX000000000.1) and *S. rubidaea* AV10 (GeneBank: JAQOZY000000000.1) were deposited in NCBI (BioProject: PRJNA925836). BioSample IDs SAMN32811356 and SAMN32811374 correspond to *S. rubidaea* EV23 and *S. rubidaea* AV10, respectively.
• This paper does not report original code
• Any additional information required to reanalyze the data reported in this paper is available from the lead contact upon request.

## Acknowledgments

R.R.V. acknowledges support from the 10.13039/501100001862Swedish Research Council for Environment, Agricultural Sciences and Spatial Planning (FORMAS; grant nos. 2019–01316), 10.13039/501100009708Novo Nordisk Fonden (0074727), 10.13039/501100004360SLU Center for Biological Control, the 10.13039/501100004359Swedish Research Council (2019–04270), 10.13039/501100004360SLU Partnerskap Alnarp and Carl Tryggers Stiftelse för Vetenskaplig Forskning (CTS 20: 464). S.C.W. acknowledges funding from the 10.13039/100012095Scottish Government Rural and Environment Science and Analytical Services (10.13039/100011310RESAS) Division (JHI-B1-1; JHI-A1-2).

## Author contributions

Conceptualization, B.G.K. and R.R.V.; methodology, B.G.K.; investigation, B.G.K. and F.G.; formal analysis, B.G.K. and S.G.; resources, R.R.V.; writing – original draft, B.G.K.; writing – reviewing and editing, B.G.K., S.G., F.G., V.C., S.C.W., R.O., and R.R.V.; funding acquisition, R.R.V.; supervision, R.R.V., and S.C.W. All authors contributed to this research work and approved the manuscript for publication.

## Declaration of interests

The authors declare no competing interests.

## Declaration of generative AI and AI-assisted technologies in the writing process

The authors did not use any AI-assisted technology to prepare this work.

## STAR★Methods

### Key resources table

**Table:** 

REAGENT or RESOURCE	SOURCE	IDENTIFIER
**Bacterial and *Phytophthora infestans* strains**		

*Serratia rubidaea* AV10	Our laboratory strain	AV10
*Serratia rubidaea* EV23	Our laboratory strain	EV23
*Pseudomonas salmasensis* SLU99	Our laboratory strain	SLU99
*Serratia proteamaculans* S4	Neupane et al.^80^	S4
*Serratia plymuthica* AS13	Neupane et al.^81^	AS13
*Phytophthora infestans* 88069	Originated from WUR, NL	88069

**Chemicals, peptides, and recombinant proteins**		

Luria-Bertani broth	Duchefa Biochemie	Product#L1704.2500
Ampicillin sodium	Duchefa Biochemie	Product#A0104.0025
Natamycin (Pimaricin)	Molekula	Product#10237077
Sucrose	Duchefa Biochemie	Product#S0809.5000
Bacto agar	Saveen & Werner	Lot#A02700
Trypan blue	Sigma Aldrich	CAS#7257-1
PBS	AppliChem GmbH	Lot#5R016944
Sodium Carboxymethyl cellulose	Sigma-Aldrich	CAS#9004-32-4
Glycerol	Duchefa Biochemie	Product#G1345.5000
Absolute ethanol	VWR chemicals	CAS#64-17-5
Revus	Syngenta	Reg#4843
Ranman Top	Certis Belchim	Reg#4995

**Critical commercial assays**		

Nanobind CBB Big DNA kit	PacBio	Cat#102-301-900
Illumina TruSeq PCR-free kit	Illumina	Cat#20015962
RNeasy® Plant Mini Kit	Qiagen	Cat#74904
qScript™ cDNA Synthesis Kit	Quantabio	Cat#95047-100
Accuris qMax™ Green qPCR mix with blue tracking dye	Accuris life science reagents	Cat#PR2000-N/LH-100
Ambion™ nuclease free water	Invitrogen	Lot#2308564

**Deposited data**		

EV23 assembled genome & raw illumina read	NCBI BioProject: PRJNA925836	GeneBank: JAQOZX000000000.1
AV10 assembled genome & raw illumina read	NCBI BioProject: PRJNA925836	GeneBank: JAQOZY000000000.1

**Oligonucleotides**		

GGGGATCTTCGTGTTGGAGTT	Bai et al.^53^	*StARF2-*FP
CCAAGCTGTTGCAAGTACACC		*StARF2*-RP
GCAGCTGTTAACTTTGGCCAA		*StLOX2.1*-FP
CCACTCCCATTCTTCAGCTGT		*StLOX2.1*-RP
TGACAAAGTGAGTGGCCTTCA		*StACO1*-FP
CCTCAAGTTGGTCACCAAGGT		*StACO1*-RP
TATTGGCTGCACGAAGTCAGT		*StNPR1*-FP
CGCACCAAATCCTTCAGCAAA		*StNPR1*-RP
TTTGCTGGTGATGATGCTCCT		*StActin7*-FP
AGCTTCATCACCCACATAGGC		*StActin7*-RP

**Software and algorithms**		

FastQC	Andrews et al.^82^	FastQC
FastP	Chen et al.^83^	FastP
SPAdes v.3.15.5	Bankevich et al.^84^	SPAdes
EzBioCloud	Yoon et al.^85^	EzBiocloud
MeDuSa	Bosi et al.^86^	MeDuSa
CheckM v.1.2.2	Parks et al.^87^	CheckM
QUAST	Gurevich et al.^88^	QUAST
PGAP	Tatusova et al.^89^	PGAP
GTDB-Tk v.2	Chaumeil et al.^90^	GTDB-Tk
DNA Plotter	Carver et al.^91^	DNA Plotter
R x64 4.2.0	Team R Core^92^	R (programming language)
ImageJ NIH software	Schneider et al.^93^	ImageJ

### Experimental model and study participant details

#### *Phytophthora infestans* and culture medium

*Phytophthora infestans* strain 88069,^94^ originally obtained from Wageningen University Research (WUR), the Netherlands, was used throughout the experiment as the source of plugs and sporangia. Rye agar (RA) was prepared by simmering 60 g L^-1^ rye grains overnight in sterilized distilled water (dH_2_O) in the dark. The next day, the grains that had simmered overnight were added to a blender (Electrolux Explore 6, blender E6TB1-4GB, China) and macerated at high speed for 1 minute. Then it was cooked in a water bath at 60°C for 3 h, after which the supernatant was filtered through multi-layer cheesecloth. The filtered supernatant was then made up to a volume of 1 L with dH_2_O, and sucrose was added to a concentration of 20 g L^-1^. The pH of the RA was adjusted to 5.8, after which agar was added to a final concentration of 15 g L^-1^ and the medium was autoclaved. After autoclaving, the media were allowed to cool to around 45°C and supplemented with ampicillin and pimaricin to final concentrations of 100 μg mL^-1^ and 10 μg mL^-1^, respectively, before being used to culture *P. infestans*.

#### Bacterial strains and growing conditions

Five bacterial strains were used in this study. Of these, *Serratia plymuthica* AS13 and *S. proteamaculans* S4 are known to promote oilseed rape plant growth and inhibit various pathogens.^80^^,^^81^ The remaining three strains (*Pseudomonas salmasensis* SLU99, *S. rubidaea* AV10, and *S. rubidaea* EV23) were isolated from the rhizosphere of potato and tomato plants in Sweden and have been used in previous studies.^57^ The SLU99 strain, previously described as *Pseudomonas fluorescens*,^46^ was reclassified as *Pseudomonas salmasensis* based on genomic analysis. Because of its previously demonstrated plant growth-promoting activity, this strain was included in the current study as a reference for comparison. All strains used in this study were routinely grown in Luria-Bertani broth (LB) (25 g L^-1^) (Sigma-Aldrich, Taufkirchen, Germany) at 28°C under shaking at 220 rpm.

### Method details

#### Field trials

A field experiment was conducted to evaluate the biocontrol potential of bacterial strains (AV10, EV23, S4, AS13, and SLU99) and to identify strains capable of reducing late blight in susceptible potato plants (cv. Desiree) under natural conditions where multiple biotic and abiotic factors influence disease development. Bacterial suspensions were prepared by growing each strain in LB broth at 28°C for 16 hours, followed by centrifugation at 4200 × g for 20 minutes at 4°C. The supernatant was then discarded, and the cell pellets were resuspended in 1× PBS (Thermo-Scientific, Waltham, MA, USA) and adjusted to an optical density (OD_600_) of 0.3. To enhance bacterial adherence to leaf surfaces, 1% carboxymethyl cellulose was added to the suspension. The experiment used a randomized complete block design (RCBD) with plots spaced 0.5 m apart and blocks spaced 1 m apart. Four blocks of seven treatments were used: 1 – Control plant, 2 - Fungicide-treated plant, 3 - S4-treated plant, 4 - SLU99-treated plant, 5 - AV10-treated plant, 6 - EV23-treated plant, and 7 - AS13-treated plant. Each treatment was replicated four times [4 rows/plot with 10 plants/row], and the area of each experimental plot was 150 m^2^ (10m wide × 15m long).

A bacterial suspension was sprayed onto plants starting 14 days after planting (1 L per plot), with four applications approximately 10 days apart over eight weeks using a backpack pressure sprayer. Control plants were sprayed only with 1x PBS supplemented with 1% carboxymethyl cellulose. Chemical fungicides were mixed and applied four times, alternately, according to the manufacturer’s instructions (Revus, 0.6 L/ha [Syngenta]; Ranman Top, 0.5 L/ha [Certis Belchim]). The trial was conducted in a farmed field in Mosslunda, Sweden (55°58′00.3″ N 14°07′03.0″ E). The average daily temperature and monthly precipitation during the study were 15°C and 53 mm, respectively. Late blight spots were recorded at 21, 30, 35, 42, and 52 days after planting by counting pathogen spots in each block. The mean disease intensity (MDI) was calculated as follows and was used because it provides a direct quantitative estimate of disease severity, particularly where disease development varies among plants and blocks.

$$\text{MDI}=\frac{\text{Number}\;\text{of}\;\text{pathogen}\;\text{spots}\;\text{in}\;\text{each}\;\text{block}}{\text{Total}\;\text{number}\;\text{of}\;\text{blocks}}$$

#### *In vitro* dual culture assay

The antagonistic potential of the bacterial strains (AS13, S4, AV10, EV23, and SLU99) against *P. infestans* was evaluated individually, as well as in compatible consortia showing synergistic potential (AS13 + EV23, AS13 + AV10, AS13 + SLU99, S4 + EV23, S4 + SLU99, EV23 + AV10, EV23 + SLU99, and AV10 + SLU99)^57^ using an *in vitro* dual culture confrontation assay. In each assay, a 5-mm mycelial plug of *P. infestans* was taken from a 2-week-old culture and placed at the center of a fresh 9-cm-diameter RA plate. Each bacterial strain was cultured overnight in 10 mL LB broth at 28°C, and the optical density at 600 nm (OD_600_) was adjusted to 0.3 for single strains or 0.15 for combined strains. These bacterial strains were streaked on both sides of the mycelial plugs, after which the plates were sealed with parafilm and incubated at 20°C. Control plates containing only the pathogen mycelial plug, with no bacteria, were also prepared. Colony diameters were measured for both control and bacteria-inoculated plates when pathogen growth reached the edge of the control plate. The percentage of mycelial growth inhibition (MGI) was calculated as follows.

MGI(%)=((R1−R2)/R1))∗100

Where *R*_1_ is the diameter of the pathogen’s mycelial growth with no antagonist, and *R*_2_ is the mycelial growth diameter with an antagonist.^95^ The antagonism assay was repeated twice with three replicates each. Mycelial growth inhibition data collected from both independent assays were pooled for statistical analysis.

#### Detached leaf assay (DLA)

Bacterial strains that strongly reduced the pathogen’s mycelial growth *in vitro* were selected for further evaluation on detached potato leaves. Briefly, disease-free potatoes (cv. Desiree) of similar sizes were planted in 2-liter plastic pots filled with sterilized compost (Krukvåxtjord Lera and Kisel, Sweden) and grown in a greenhouse at 20°C with 72% RH. The plants were rotated weekly and watered three times per week. Fully expanded 3^rd^ and 4^th^ leaves of approximately the same age were detached from the middle sections of four-week-old potato plants, washed with sterile distilled water, and placed adaxial side up on moistened filter paper in plastic boxes. Bacterial strains cultured overnight were centrifuged at 4200 × g for 20 minutes at 4°C. The collected bacterial cells were resuspended in 1× PBS, and the OD_600_ was adjusted to 0.3 for single strains and 0.15 for mixed strains.

A 10 μL aliquot of bacterial suspension (or 1× PBS for control leaves) was then inoculated onto leaves one day before pathogen inoculation. The following day, *P. infestans* sporangia were collected from two-week-old agar plate cultures by flooding them with sterile dH_2_O, rubbing them with a sterile spreader, and filtering through a sterile 40 μm cell strainer (Starlab, Germany). The sporangia concentration was adjusted to 2.5 × 10^4^ sporangia/mL using a 0.2 mm hemacytometer (Fuchs-Rosenthal, Germany), after which 10 μL of the sporangia suspension was spot-inoculated onto the leaves at the location where the bacterial suspension had been applied. The inoculated leaves were incubated at 22°C and 72% RH for 6 days.

After the incubation period, the leaves were immersed in trypan blue solution for 30 minutes,^96^ followed by overnight incubation in absolute ethanol (99.7%) at room temperature.^97^ Ethanol-de-stained leaves were immersed in a 30% glycerol solution for 30 minutes. Images were taken using an Epson Perfection V750 Pro scanner (model J221A 24V, Indonesia), and the leaves’ necrotic areas were quantified using ImageJ NIH software.^93^ This assay was performed with five replicates per treatment and was repeated twice. Necrotic lesion data from both independent assays were pooled for statistical analysis.

#### Greenhouse trials

The individual strains and consortia providing the smallest proportional leaf lesion areas in the detached-leaf assays were selected for further evaluation of their disease-protective effects under greenhouse conditions. For this experiment, the chosen strains were grown overnight and then centrifuged at 4200 × g for 20 minutes at 4°C. The OD_600_ was adjusted to 0.3 for single strains or 0.15 for consortia in 1× PBS. The suspensions were then used to soak uniformly sized tubers for one hour. Control tubers were instead soaked with 1× PBS. Tubers treated with bacterial cells were air-dried at room temperature for 30 minutes, then planted in 1.5 L pots filled with sterilized compost (Krukvåxtjord Lera and Kisel, Sweden) and grown in a greenhouse at 20°C and 65% (RH) for four weeks. Two weeks after planting, suspensions of the same bacterial cells with the same OD were applied around the root zone of each potato plant (20 mL/pot). The plants were rotated weekly during the day and watered three times a week. In the fourth week, the appropriate bacterial suspensions (with the same OD as before) were sprayed onto potato leaves with a hand sprayer (20 mL/pot), 1 day before pathogen inoculation. Finally, six leaflets per pot were spot-inoculated with 10 μL of *P. infestans* sporangia (2.5× 10^4^ spores/mL).

The pathogen-inoculated plants were covered with a plastic sheet to increase humidity and maintained at 20°C with 85% RH to facilitate infection. The progression of pathogen infection was monitored daily. Disease symptoms were assessed quantitatively 6 days after pathogen inoculation, using a slightly modified protocol^98^ in which infected leaf area is graded on a rating scale ranging from 0 to 4 [0 = no visible symptoms; 1 = 0.1- 25% of the total leaf area infested; 2 = 25.1-50% of the total leaf area infested; 3 = 50.1-75% of the total leaf area infested; 4 > 75.1% of the total leaf area infested]. The disease severity index (DSI%) for each disease rating was calculated as follows: DSI% = 100 × (sum of numerical ratings for the treatment) / (No. plants examined × maximum disease scale).^99^ The experiment was performed using a randomized complete design with four replicates per treatment, each replicate consisting of six leaflets.

#### Genomic DNA isolation and sequencing

The genomes of *S. rubidaea* AV10 and *S. rubidaea* EV23 were sequenced because they represented new strains with promising, distinct levels of biocontrol and plant growth-promotion potential, to identify genes potentially involved in disease suppression and plant growth promotion. Strains AV10 and EV23 were grown in LB broth at 28°C for 18 hours, and their genomic DNA was extracted using a Nanobind CBB Big DNA kit (Circulomics Kit, Baltimore, MD, USA) following the manufacturer’s instructions. DNA concentration was measured using a NanoDrop DS-11FX spectrophotometer/fluorometer (DeNovix product, USA), and the quality and integrity of the extracted DNA were assessed by gel electrophoresis on a 1% agarose gel. DNA libraries for both strains were generated with an Illumina TruSeq PCR-free kit (Thermo Scientific, Waltham, MA, USA) and sequenced on a MiSeq platform using MSC 2.5.0.5/RTA 1.18.54 software, employing 2 × 300 bp paired-end reads at SciLifeLab, Sweden.

#### Genome assembly, evaluation, and annotation

The quality of all Illumina reads was assessed using FastQC,^82^ and FastP^83^ was used for quality control and read preprocessing. The high-quality, filtered reads from both strains were assembled separately using SPAdes v.3.15.5.^84^ The assembled primary genome fasta and the closest reference for the primary assembled genome, as identified by EzBioCloud,^85^ were used for assembly polishing. The contigs of the primary assembled genome were arranged into a scaffold using the web-based tool MeDuSa.^86^ The completeness, contamination, and lineages of assembled genomes were assessed using CheckM v.1.2.2.^87^ QUAST^88^ was used to assess the genome assemblies by computing metrics including N50, L50, size, and GC%.

The NCBI Prokaryotic Genome Annotation Pipeline (PGAP)^89^ was used for gene prediction and annotation. GTDB-Tk v.2^90^ was used for taxonomic assignment of the annotated genomes. Circular maps showing genomic features were generated with DNA Plotter.^91^ Average nucleotide identity (ANI) and average amino-acid identity (AAI) were calculated using Pyani^100^ and CompareM,^101^ respectively. Rapid Annotation using Subsystem Technology (RAST)^102^ was used to annotate the subsystem categories. Similarly, COG classifier v1.0.5^103^ was used to annotate clusters of orthologous groups (COGs). Both annotations were visualized in a heatmap using the R package “pheatmap”.^104^ The web-based antiSMASH 7.0 bacterial version^105^ was used to identify genes associated with secondary metabolites. GO terms obtained from the PGAP^89^ annotation were grouped using CateGOrizer.^106^ Pathways were annotated with BlastKOALA.^107^ Core genomes were generated using OrthoVenn3.^108^ Genes associated with plant growth-promoting traits (PGPTs) and plant interaction factors (PIFRs) were identified using the PlaBAse resource.^109^

#### Phylogenetic tree construction

The UBCGv2 pipeline^110^ was used to build a phylogenetic tree based on a predefined bacterial core gene set comprising 81 core genes from 3,508 qualified species. Tree construction was performed using all available genomes for the species under investigation and one additional species (outgroup) from NCBI GenBank. Finally, the tree file was converted to the Newick format for visualization in iTOLv5.^111^

#### Plant growth parameters

To investigate the possible role of these strains in promoting plant growth, similarly sized potato tubers were soaked in the same bacterial cell suspension adjusted to the same OD_600_ used in the plant infection assays. Treated tubers were then planted in a greenhouse maintained at 20°C and 65% RH, and grown for four weeks under the previously described conditions, with 8 replicates per treatment. Two and three weeks after planting, the same bacterial cell suspensions were applied to the root zone of the corresponding potato plants (20 mL per pot). Tubers treated with 1x PBS only served as the control. At the end of the four-week growth period, potato growth parameters were assessed by measuring plant height (cm), chlorophyll content (μmol m^-2^), fresh weight (g), dry weight (g), and root weight (g).

#### Relative expression of growth-related and ISR signaling genes

Potato tubers of similar size were soaked in suspensions of AV10 and EV23 strains, which have putative plant growth-promoting traits and induce systemic resistance signaling pathways, using the same OD_600_ as previously stated. Treated tubers were then planted in the greenhouse under the conditions described earlier and grown for four weeks. Two weeks after planting, the same bacterial strains were applied to the root zone of the corresponding potato plants (20 mL per pot). At the end of four weeks, *P. infestans* was spot-inoculated, and leaf samples were collected at 12, 24, 48, and 72 hours post-inoculation. Using a Mixer Mill MM 400 (RETSCH, Germany), collected leaf samples were ground with stainless steel beads at 20 Hz for 3 minutes under frozen conditions. After homogenization, total RNA was extracted using the RNeasy Plant Mini Kit (Qiagen, Hilden, Germany) according to the manufacturer’s instructions. One microgram of RQ1 RNase-free DNase-treated RNA was reverse transcribed using the qScript cDNA Synthesis Kit (Quantabio, Beverly, MA, USA) on a PCR instrument at 22°C for 5 min, 42°C for 30 min, and 85°C for 5 min. The cDNA sample was diluted 2-fold as the template for RT-qPCR amplification. The relative expression of potato genes associated with growth promotion, such as auxin response factors (*StARF2*) and jasmonic acid and ethylene biosynthesis genes, such as Lipoxygenase (*StLOX2.1*) and 1-aminocyclopropane-1-carboxylate oxidase (*StACO1*), as well as resistance regulatory genes, such as nonexpressor of pathogenesis-related genes 1 (*StNPR1*), was detected. The *StActin7* gene was used as the control. All primer sequences used for RT-qPCR are listed in the key resources table (see STAR Methods). Three biological replicates with three technical replicates were used for each sample run using Accuris qMax Green qPCR mix with blue tracking dye (Edison, NJ 08818, USA). For each gene, the delta delta Ct (2^−ΔΔCT^) expression value was calculated as in an earlier study.^112^

### Quantification and statistical analysis

A field experiment was conducted using four independent replicate plots per treatment (*n* = 4), each plot containing four rows of 10 plants (40 plants per plot). Disease spots were recorded over time, and mean disease spot counts per plot were calculated and used for statistical analysis. *In vitro* mycelial growth inhibition and detached leaf assays were carried out in two independent experimental repetitions, each consisting of three (*n* = 3) and five (*n* = 5) replicates, respectively. Linear mixed-effects models, with treatment as a fixed effect and experimental repetition as a random effect, were used to analyze data from both *in vitro* and detached leaf assays. Treatment means were compared using Duncan's multiple range test (DMRT) at *p ≤ 0.05*. In the greenhouse, each treatment had four biological replicates (*n* = 4), each consisting of six leaflets. The single averaged value obtained from six leaflets for each biological replicate was analyzed using one-way analysis of variance (ANOVA), with treatment as a fixed effect, followed by mean comparison using DMRT at *p ≤ 0.05*. For gene expression experiments, each treatment group consisted of three biological replicates (*n* = 3), with three technical replicates for each biological replicate. A single value was obtained by averaging the technical replicates for each biological replicate before statistical analysis. Subsequently, data were analyzed using one-way ANOVA, followed by mean comparison by DMRT at *p ≤ 0.05*. For all experiments, field, *in vitro*, detached leaf, and greenhouse data are presented as mean ± SD, and different letters indicate significant differences between treatments. All statistical analyses were performed using R x64 4.2.0 software.^92^ All bioinformatics-related analyses were conducted as described in the STAR Methods section.

## Supplemental information

Document S1. Figures S1–S5

Table S1. Genome statistics (Quast, CheckM, ANI, and AAI) of *S. rubidaea* EV23, *S. rubidaea* AV10, and selected strains, related to Figures 2A, 2B, and 2C

Table S2. OrthoVenn analysis of *S. rubidaea* AV10, *S. rubidaea* EV23, and *S. rubidaea* FDAARGOS926, related to Figure 2D

Table S3. Gene Ontology analysis of *S. rubidaea* AV10, *S. rubidaea* EV23, and *S. rubidaea* FDAARGOS926, related to Figure 3A

Table S4. KEGG pathway analysis of *S. rubidaea* AV10, *S. rubidaea* EV23, and *S. rubidaea* FDAARGOS926, related to Figure 3B

Table S5. Plant growth-promoting traits of *S. rubidaea* AV10, *S. rubidaea* EV23, and *S. rubidaea* FDAARGOS926, related to Figures 3C, 3D, and Table 3

Table S6. Plant-interacting factors of *S. rubidaea* AV10, *S. rubidaea* EV23, and *S. rubidaea* FDAARGOS926, related to Figures 3E, 3F, and Table 3
