# Supplementary material for: Rhizobacterial biocontrol of potato late blight and genomic insights into disease suppression and growth promotion
Source: iScience. 2026 Sep 22;29(10):117682. doi: 10.1016/j.isci.2026.117682 (PMC13675477; doi:10.1016/j.isci.2026.117682)
Supplement: Supplementary file 1 [file mmc1.pdf]

iScience, Volume *29*

Supplemental information

Rhizobacterial biocontrol of potato late blight and genomic insights into disease suppression and growth promotion

Bekele Gelena Kelbessa, Samrat Ghosh, Farideh Ghadamgahi, Vittoria Catara, Stephen C. Whisson, Rodomiro Ortiz, and Ramesh Raju Vetukuri

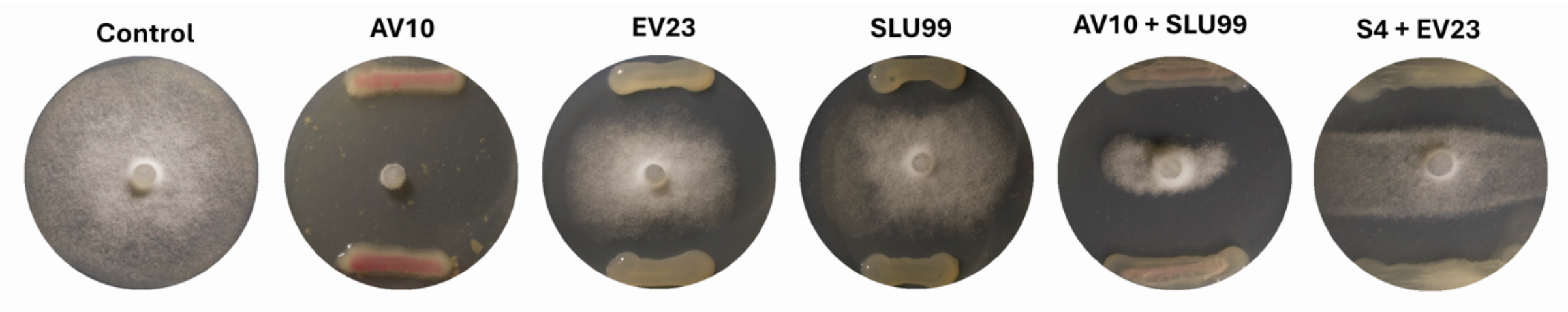

Figure S1. Bacterial strains exhibit antagonistic activity against *P. infestans*, suppressing pathogen mycelial growth relative to the control on rye agar plates, related to Figure 1B.

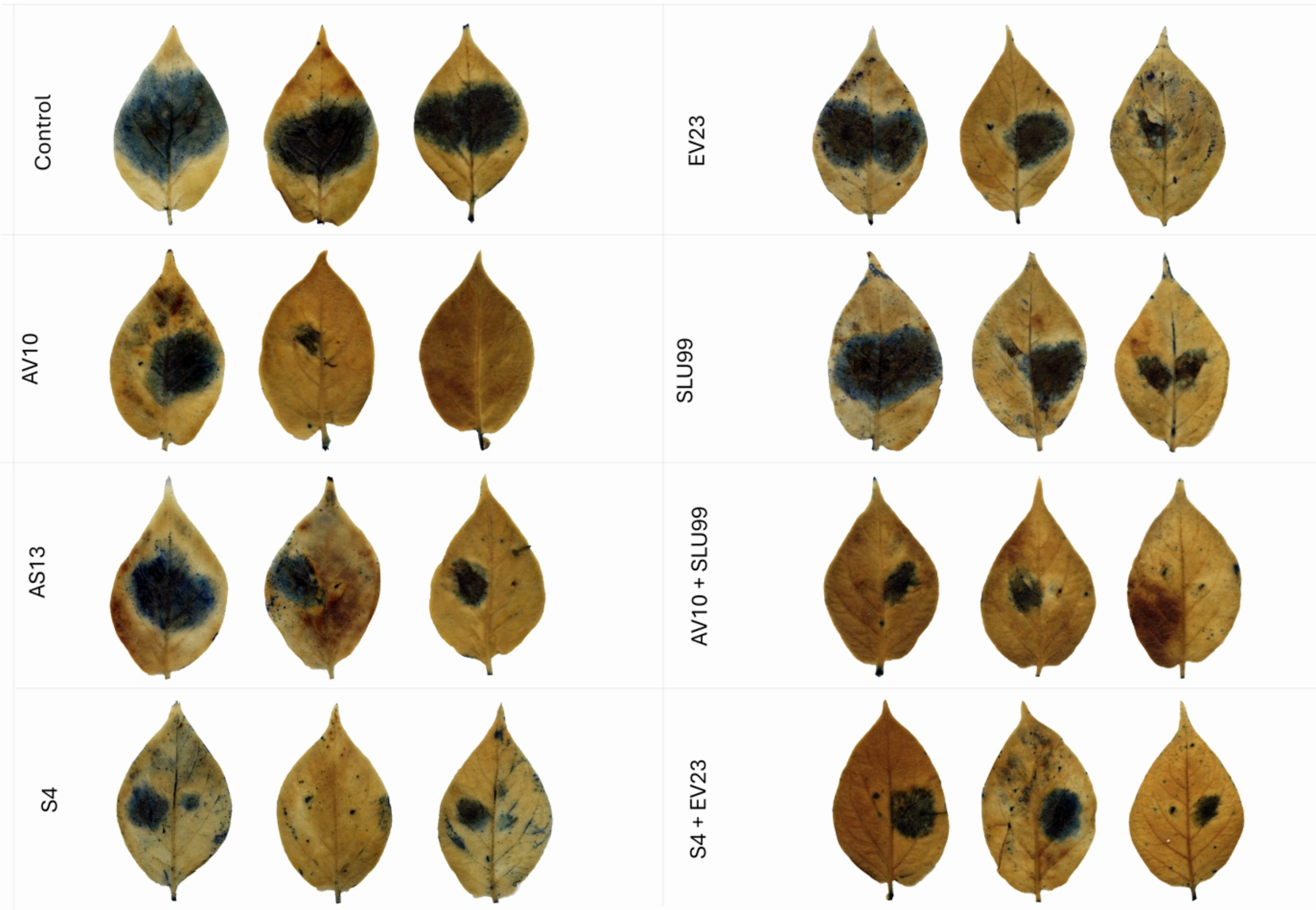

Figure S2. Trypan blue staining of detached potato leaves was used to assess the biocontrol potential of bacterial strains against *P. infestans*, related to Figure 1C. The positive infection control showed extensive trypan blue staining extending beyond the inoculation sites, indicating strong pathogen infection. Treatments with selected bacteria resulted in limited *P. infestans* infection, as evidenced by minimal trypan blue staining beyond the initial inoculation site.

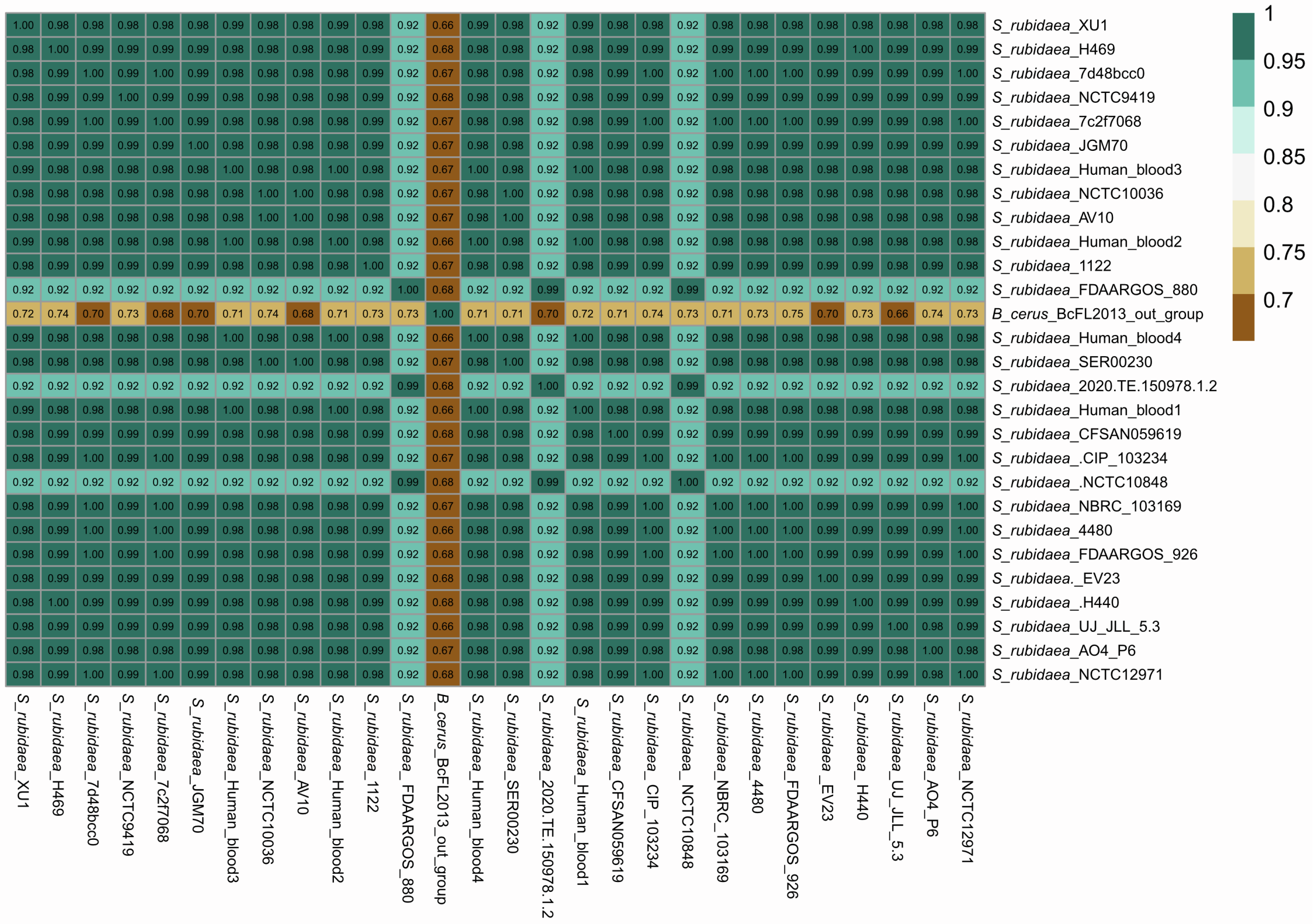

Figure S3. Average Nucleotide Identity (ANI) of *Serratia rubidaea* strains, related to Figure 2C. Heat maps show the variation in Average Nucleotide Identity (ANI) for *Serratia rubidaea* strains. The values scale shows nucleotide-based percent similarity, with brown representing low identity and green representing high identity.

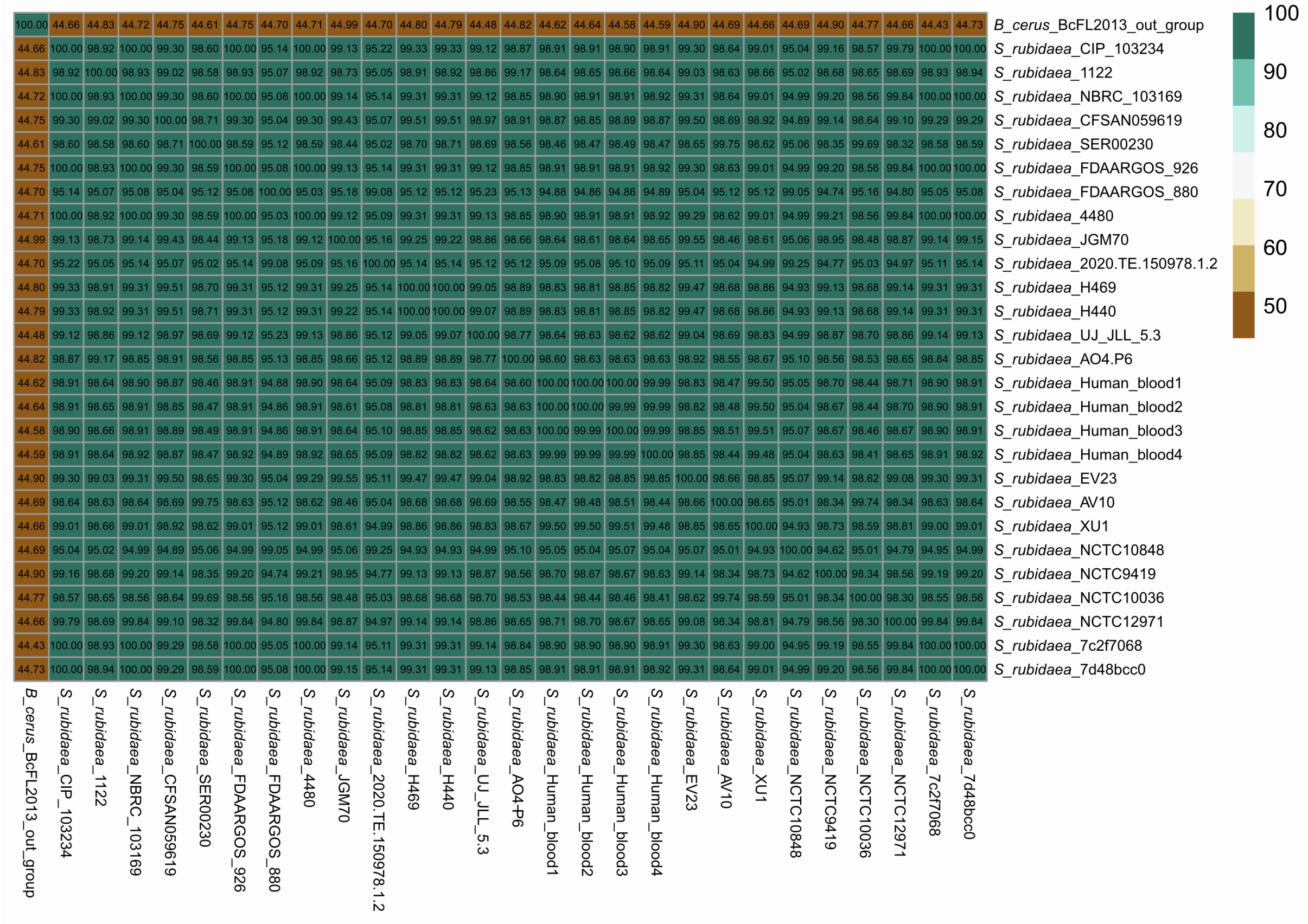

Figure S4. Average Amino Acid Identity (AAI) of *Serratia rubidaea* strains, related to Figure 2C. Heat maps show the variation in Average Amino Acid Identity (AAI) for *Serratia rubidaea* strains. The values scale shows percent similarity based on amino acid sequences, with brown indicating low identity and green indicating high identity.

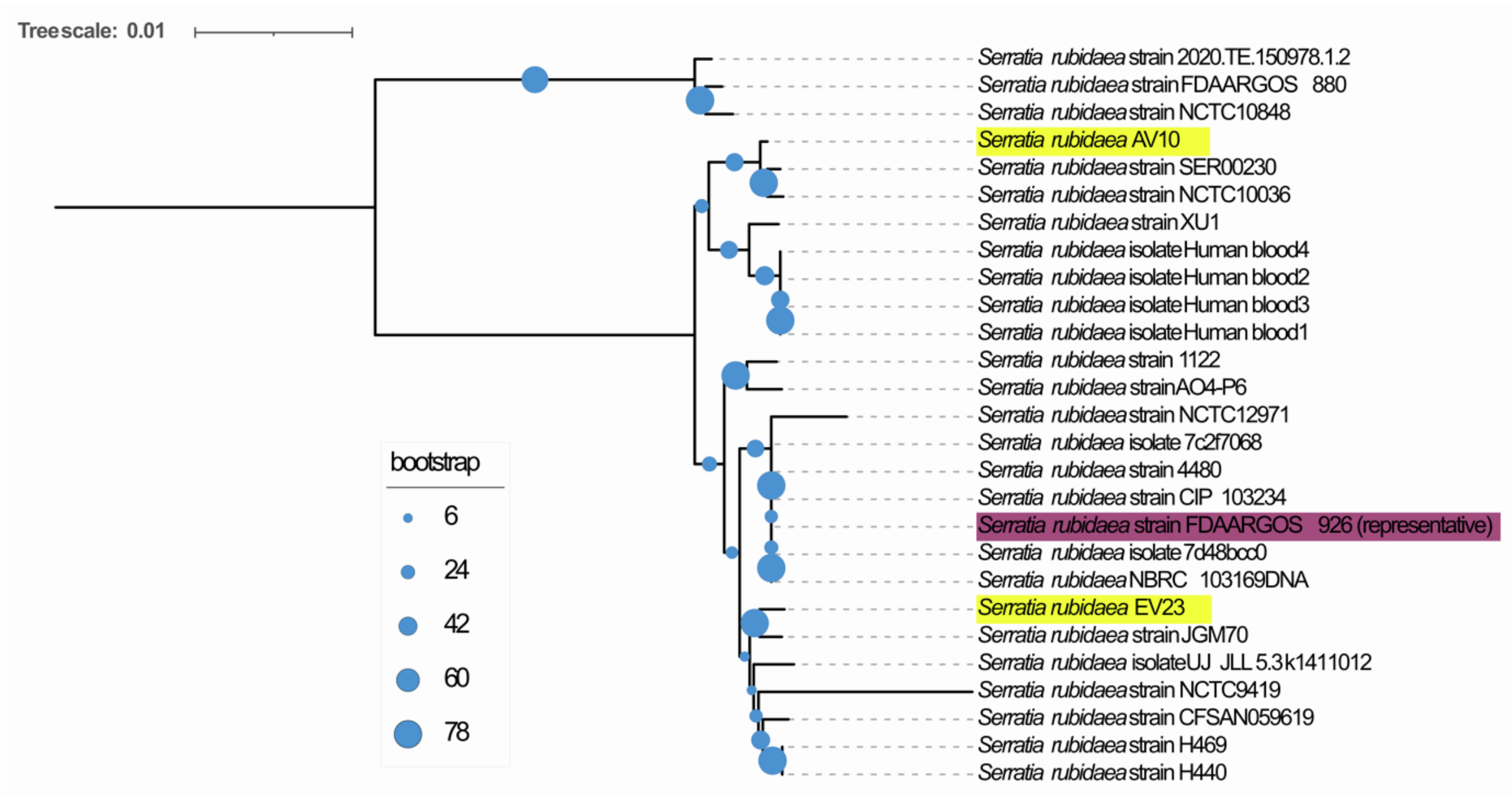

Figure S5. Phylogenetic tree based on 81 bacterial core genes, showing the relationship of *S. rubidaea* AV10 and *S. rubidaea* EV23 (in yellow) to other *S. rubidaea* strains in a tree without an outgroup, related to Figure 2C.
